# Pain pressure threshold of a muscle tender spot increases following local and non-local rolling massage

**DOI:** 10.1186/s12891-015-0729-5

**Published:** 2015-09-28

**Authors:** SJ Aboodarda, AJ Spence, Duane C. Button

**Affiliations:** 1 School of Human Kinetics and Recreation, Memorial University of Newfoundland, 230 Elizabeth Avenue, St. John’s, Newfoundland A1C 5S7 Canada; 2 Faculty of Medicine, Memorial University, St. John’s, Newfoundland and Labrador Canada

**Keywords:** Roller massage, Perceived pain, Plantar flexors, Tender spot, Algometry

## Abstract

**Background:**

The aim of the present study was to determine the acute effect of rolling massage on pressure pain threshold (PPT) in individuals with tender spots in their plantar flexor muscles.

**Methods:**

In a randomized control trial and single blinded study, tender spots were identified in 150 participants’ plantar flexor muscles (gastrocnemius or soleus). Then participants were randomly assigned to one of five intervention groups (*n* = 30): 1) heavy rolling massage on the calf that exhibited the higher tenderness (Ipsi-R), 2) heavy rolling massage on the contralateral calf (Contra-R), 3) light stroking of the skin with roller massager on the calf that exhibited the higher tenderness (Sham), 4) manual massage on the calf that exhibited the higher tenderness (Ipsi-M) and 5) no intervention (Control). PPT was measured at 30 s and up to 15 min post-intervention via a pressure algometer.

**Results:**

At 30 s post-intervention, the Ipsi-R (24 %) and Contra-R (21 %) demonstrated higher (*p* < 0.03) PPT values compared with Control and Sham. During 15 min post-intervention, PPT was higher (*p* < 0.05) following Ipsi-R (19.2 %), Contra-R (15.9 %) and Ipsi-M (10.9 %) compared with Control. There was no difference between the effects of three deep tissue massages (Ipsi-R, Ipsi-M and Contra-R) on PPT.

**Discussion:**

Whereas the increased PPT following ipsilateral massage (Ipsi-R and Ipsi-M) might be attributed to the release of fibrous adhesions; the non-localized effect of rolling massage on the contralateral limb suggests that other mechanisms such as a central pain-modulatory system play a role in mediation of perceived pain following brief tissue massage.

**Conclusion:**

Overall, rolling massage over a tender spot reduces pain perception.

**Trial registration:**

ClinicalTrials.gov (NCT02528812), August 19^th^, 2015.

## Background

Myofascia has been defined as “a dense irregular connective tissue that surrounds and connects every muscle, even the tiniest myofibril, and every single organ of the body” [1]. This system is thought to be responsible for facilitation of mobility, cellular circulation and elasticity of muscle tissues. Myofascia may contract and bond to the neighboring structures in response to injury, postural stress, and inactivity [1, 2]. Since the fascia is densely innervated by sensory neurons (i.e. free nerve endings [nociceptors] which function as pain receptors) myofascial adhesions may create “hypersensitive tender spots” [2, 3]. Physiotherapeutic sensory stimulation such as massage over these tender spots may be an effective complementary treatment for pain alleviation [4]. Massage-like mechanical pressure may potentiate analgesic effects neurologically (e.g. mediation of pain-modulatory system), physiologically (e.g. increase blood and parasympathetic circulation) and mechanically (e.g. rearrangement of muscle fibers, connective tissue and blood vessels) [4–8].

One therapeutic mode of massage that is frequently used for treatment of pain is myofascial release [1, 2, 9, 10]. Among various myofascial release techniques, self myofascial release using foam rollers and roller massagers have been increasingly practiced in clinical and athletic settings to promote soft-tissue extensibility and optimal muscle functioning, subsequently reducing pain [11–14]. Foam rolling technique requires individuals to use their bodyweight during rolling a specific body region over a dense foam cylinder; whereas during self massage using hand-held roller massager, upper body strength (rather than body weight) is employed to exert pressure on muscle tissue [15]. Rolling massage has demonstrated positive effects on arterial dilation and vascular plasticity [8], plasma nitric oxide concentration [8], reduction of sense of fatigue [12], increased range of motion [15], connective tissue recovery following exercise-induced muscle soreness [13, 16], and increased neuromuscular efficiency [17]. Based on the aforementioned studies, it is plausible that rolling massage may alter how an individual perceives pain.

Three studies have shown decreases in pain perception due to foam rolling. Vaughan and McLaughlin [14] demonstrated that 3-min of foam rolling over the iliotibial band resulted in significant increase in pressure pain threshold (PPT) immediately post-treatment. Macdonald et al. [13] and Pearcey et al. [16] studied the effect of foam rolling on recovery from exercise-induced delayed onset of muscle soreness (DOMS) and found that foam rolling reduced pain perception throughout the period of DOMs. These investigators speculated that foam rolling might reduce pain perception via restoration of soft tissue extensibility and/or activation of a central pain-modulatory system. However, there is little research investigating the effects of rolling massage on pain perception in individuals with “hypersensitive tender spots” or if the increased PPT following rolling massage is mainly due to restoration of the soft-tissue and central pain-modulatory system.

PPT measurement using pressure algometry has been suggested as an accurate, valid and reproducible method for diagnosis of tender spots and assessment of treatment results [18–22]. However, the average of more than one PPT measurement at a site was recommended for a better estimation of the relative tenderness [23]. Several investigations have reported high reliability coefficients (range: 0.71–0.97) for 2 to 5 repeated PPT algometry trials over tender spots in various muscle groups [19, 22, 23]. Wolff and Jarvik [24] have suggested to discard the first trial of pain threshold measurement and use the average of at least 5 trials for heat, cold and chemical stimulations. Using different protocols of repeated PPT trials (with different rest interval between trials) and testing various muscle groups with different amounts of tenderness warrant further investigations to prove the reliability of repetitive PPT algometry on hypersensitive tender spots in plantar flexor muscles.

Therefore, the aim of the present study was to determine the immediate effects of rolling massage on PPT in individuals having hypersensitive taut bands in their plantar flexor muscles. Specifically, we compared the effects of heavy deep tissue rolling (on both the ipsilateral and contralateral plantar flexors) and manual massage, light rolling massage and control on PPT of the tender spot on the ipsilateral plantar flexors. We hypothesized that deep tissue massages, either using rolling massage (ipsilateral only) or manual massage, would immediately increase PPT (i.e. decrease pain perception or increase pain tolerance). A second aim of the study was to ascertain the time course of acute changes (i.e. up to 15 min following massage) in PPT values following each massage intervention. We hypothesized that increased PPT would be transient and there would no longer be an effect 15 min following the massage. The third aim was to determine the reliability of the repeated PPT measurement (i.e. PPT trials with 5–10 s intervals) performed on hypersensitive tender spots in plantar flexor muscles.

## Methods

### Participants

One hundred and fifty university-aged and recreationally active participants including 80 males and 70 females volunteered for the study. To be included, participants had to exhibit point tenderness in their plantar flexor muscles (gastrocnemius or soleus) either in the left or right limb. Tender points were identified by a registered massage therapist and defined as dense, hypersensitive areas found within a palpable taut band of muscle tissue. Participant exclusion criteria included: having musculoskeletal or visceral chronic pain and taking pain relief medications within the past 24 h. Participants who fulfilled the inclusion criteria were verbally informed of all experimental procedures and if willing to participate, read and signed a written consent form (which also had details of the experimental procedures). The Interdisciplinary Committee on Ethics in Human Research (ICEHR) of the Memorial University of Newfoundland approved the study (Approval #: 20140537-HK).

### Research design

In a randomized, single blinded, control trial study tender spots were identified in participants’ plantar flexor muscles (gastrocnemius or soleus). The acute effect of different roller massage interventions on PPT (i.e. change in PPT from pre- to post-intervention) was investigated. Participants were not informed of the intervention groups. The same registered massage therapist who was blinded to the results of the pre- and post-intervention measurements administered the different modes of massage. In addition, the researcher who recorded the PPT values at pre- and post-intervention levels was blinded to the mode of intervention administered by the massage therapist. Sisto et al. 2007 [25] has shown that the intra-day and inter-day correlations for the algometry muscle tester ranges from 0.88 to 0.99 and 0.94 to 0.98, respectively. The CONSORT guidelines were followed throughout the current research study. The study duration was from June to October 2014.

### Experimental protocol

Each participant attended the lab for one experimental session. The participants lay on a massage table in a prone position and a registered massage therapist with more than 5 years experience examined the relaxed plantar flexor muscles to identify tender spots. A tender spot was identified when an area was hyperirritable on palpation and pressure applied to the area with the pressure pain algometer (with approximately 4 kg/cm^2^) elicited localized pain greater than 5/10 on a visual analogue scale (VAS) [26]. The VAS scale was a horizontal line with anchors at the ends indicating no pain (score of 0) and intolerable pain (score of 10) [27, 28]. The identified tender spots were marked with a permanent marker and the spot that exhibited the highest value was chosen for further measurements.

Then the local tenderness (dependent variable) was quantified (by the same massage therapist who screened the participants) measuring the PPT using the pressure pain algometer. The algometer (Lafayette Manual Muscle Test System™, Model 01163, Lafayette Instrument Company, Indiana, USA) was a hand-held muscle tester with a range of 0–300 lb (136.1 kg) that consisted of a padded disc with a surface area of 1.7 cm^2^ attached to a microprocessor-control unit that measures peak force (pounds or kilograms). The unit has a digital readout for peak-applied pressure and provides a built-in calibration routine that verifies a valid calibration. In order to determine PPT, the researcher would apply the algometer to the tender spot on the participants calf muscle and increase the amount of pressure until the participant verbally informed the researcher when the sensation of pressure became pain [20, 23] at which point the algometer was removed and the PPT value was recorded. PPT values were obtained every 5–10 s over the tender spot using a pressure algometer and PPT was measured 6 times. Since the post-intervention PPT measurement was time sensitive, 6 PPT trials with 5–10 s interval were performed to gain a consistent value for this measurement (See the Results and Discussion).

After completion of pre-intervention PPT measurements, participants were randomly (using a random number generator) assigned to one of five intervention groups (*n* = 30 for each group) including 1) heavy rolling massage on the calf that exhibited the higher tenderness (Ipsilateral Rolling: Ipsi-R), 2) heavy rolling massage on the calf of the contralateral limb (Contralateral Rolling: Contra-R), 3) light stroking of the skin with roller massager on the calf that exhibited the higher tenderness (Sham), 4) manual massage on the calf that exhibited the higher tenderness (Ipsilateral Manual: Ipsi-M) and 5) no intervention (Control). The four massage intervention groups (Ipsi-R, Contra-R, Sham, Ipsi-M) involved 3 sets of 30 s massage with 30 s of rest between sets. The registered massage therapist performed the roller massage via a Theraband® roller massager (Hygienic Corporation, Akron, OH) [29] technique on the Ipsi-R, Contra-R and Sham groups. The roller massager consisted of a hard rubber material (24 cm in length and 14 cm circumference) with low amplitude, longitudinal grooves surrounding a plastic cylinder [29]. The participants were instructed to provide feedback on the level of perceived pain during the heavy rolling and manual massage (a combination of compressions and petrissage) and the intensity of massage would be adjusted accordingly to ensure 7/10 on the visual analogue scale (VAS) was maintained. The roller massager was moved proximal to distal at a slow pace (2 s up and 2 s downs) over the muscle belly. Participants in the Sham group received very light pain-free cutaneous strokes of rolling massage with the same pace of rolling as performed for the Ipsi-R and Contra-R groups. The Control group did not receive any treatment. They lay on the table in prone position for 3 min until post-intervention data was collected. The Control group was assigned in the present study to account for the potential confounding influence of cutaneous touch in Sham.

#### Primary outcome measure

**Fig. 1:**
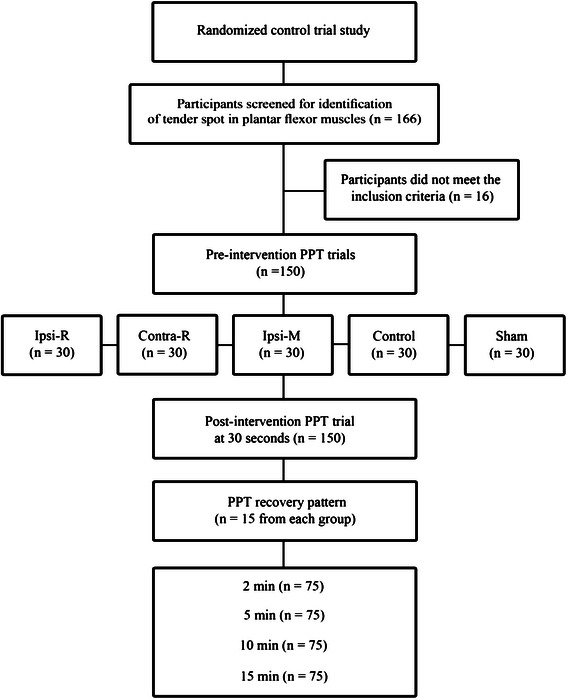
Experimental design. PPT: Pressure pain threshold, Ipsi-R: heavy rolling massage on the calf that exhibited the higher tenderness, Con-R: heavy rolling massage on the calf of the contralateral limb, Sham: light stroking of the skin with roller massager on the calf that exhibited the higher tenderness, Ipsi-M: manual massage on the calf that exhibited the higher tenderness, and Control: no intervention

Thirty seconds after completion of interventions, PPT values were obtained every 5–10 s over the tender spot and PPT was measured 6 times. In order to quantify the recovery pattern of the pain threshold following different interventions, 15 of the 30 participants from each intervention group were recruited and randomly assigned to the 5 intervention groups to undertake PPT measurements up to 15 min post-intervention (*n* = 75 participants including 40 males and 35 females). Accordingly, PPT measurements were repeated at 2, 5, 10, and 15 min following interventions (see Fig. 1 for experimental set-up). For each participant, the average of post-intervention PPT values measured at each time period was then normalized to the average of pre-intervention PPT measurements.

#### Secondary outcome measure

In order to measure the reliability of the repetitive pressure pain algometry, the intra-class correlation coefficient (ICC) [30] and coefficient of variance (CV) [31] were calculated for the 6-pre-intervention PPT measurements (*n* = 150).

### Statistical analysis

Statistical analyses were computed using SPSS software (Version 16.0, SPSS, Inc, Chicago, IL). Assumption of sphericity (Mauchley test) and normality (Shapiro-Wilk test) were tested for all of the dependent variables. If the assumption of sphericity was violated, the corrected value for non-sphericity with Greenhouse-Geisser epsilon was reported. First, a one-way repeated measure analysis of variance (ANOVA) was computed to identify the effect of 6 pressure algometry measurements (*n* = 150) on pre- and post-intervention PPTs, separately. If significant main effects were detected, a Bonferroni (Dunn’s) procedure was used to identify the significant changes over the six measurements. Due to the large number of comparisons a Bonferoni correction was used to limit the Type I error. Statistical significance was defined as *p* < 0.01. Second, in order to determine effect of the five interventions (Ipsi-R, Contra-R, Sham, Ipsi-M and Control) on normalized pain threshold at 30 s post-intervention (*n* = 150) a one-way ANOVA with Bonferroni post hoc test was used. Third, in order to determine the recovery pattern of normalized PPT values across five post-intervention time points (30 s, 2, 5, 10, and 15 min) a two-way ANOVA (5 interventions ×5 time points), with Bonferroni post hoc test was used (*n* = 75). Additionally, Cohen’s d effects sizes (ES [32]) were also calculated to determine the magnitude of the differences between interventions and time. The following criteria were used: ES <0.2 was classified as trivial, ES = 0.2–0.49 was considered as “small” effect size; ES = 0.5–0.79 represented a “medium” effect size; and ES >0.8 represented a “large” effect size. Statistical significance was defined as *p* < 0.05. The effect of different types of massage on PPT was not measured for gender due to a small sample size. Riley et al. [33] suggest that a minimum of 41 subjects per group was required for studying gender effects.

## Results

One hundred and sixty six participants were screened to identify tender spots in their plantar flexor muscles (gastrocnemius or soleus) among which 150 subjects including 80 males (26.7 ± 6.3 years, 176.2 ± 5.7 cm, 79.8 ± 15.1 kg) and 70 females (24.6 ± 6.1 years, 161.8 ± 7.7 cm, 65.4 ± 12.3 kg) met the inclusion criteria of the study. Sixteen participants were excluded from the study after initial screening because they did not have hypersensitive tender spots in their plantar flexors.

### Pressure pain threshold (PPT) reliability (*n* = 150)

**Fig. 2:**
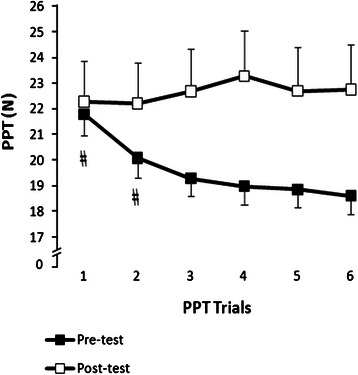
Pressure pain threshold (PPT) values (mean and SE) pre- and 30s post-intervention (*n* = 150, 30 per group). # denotes that the PPT value was significantly greater than 3rd to 6th trials (*p* < 0.01)

The ICC for the 6 pre-intervention trials was 0.93. The CV for PPT measurements 1 to 6 were 46.8, 46.4, 47.1, 46.6, 46.2 and 46.7 %, respectively (Fig. 2). The one-way ANOVA demonstrated a significant difference between the 6 pre-intervention PPT values (F = 33.95, *p* < 0.001) where, the first two PPT values were significantly higher than the 3rd, 4th, 5th and 6th values (all *p* < 0.01, ES: 0.2–0.4). No further significant differences were observed between the 3rd to 6th pre-intervention PPT values (Fig. 2). Accordingly, the average of four (3rd to 6th) PPT values was calculated as a baseline value for all groups. An average of all six would have overestimated the mean PPT. Since there was no significant difference between 6 post-intervention PPT (*p* = 0.38) values, the average of all 6 PPT values was computed as one post-intervention PPT value.

### Normalized PPT measurements at 30 s post-interventions (*n* = 150)

**Fig. 3:**
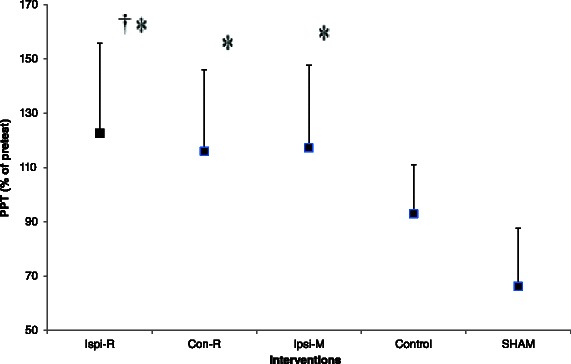
Pressure pain threshold (PPT) values (mean and SE) 30s post-intervention depicted as a percentage of pre-intervention value for each intervention group (*n* = 150, 30 per group). Ipsi-R: heavy rolling massage on the calf that exhibited the higher tenderness, Contra-R: heavy rolling massage on the calf of the contralateral limb, Sham: light stroking of the skin with roller massager on the calf that exhibited the higher tenderness, Ipsi-M: manual massage on the calf that exhibited the higher tenderness, and Control: no intervention. * denotes that the PPT was greater than Sham. † denotes that the PPT was greater than Control

A one-way ANOVA demonstrated a significant main effect for 5 interventions (F = 10.22, *p* < 0.001). Post hoc analysis demonstrated that there were significantly higher PPT values following Ipsi-R (Mean Difference [MD] = 24.6 ± 6.9 %, *p* = 0.006, ES = 0.7) and Contra-R (MD = 21.8 ± 7.0 %, *p* = 0.02, ES = 1.0) compared with the Control group. Post hoc analysis also demonstrated that there were significantly higher PPT values following Ipsi-R (MD = 39.2 ± 6.9 %, *p* < 0.001, ES = 1.1), Contra-R (MD = 36.4 ± 6.8 %, *p* < 0.001, ES = 1.5) and Ipsi-M (MD = 31.3 ± 6.7 %, *p* < 0.001, ES = 1.3) compared with the Sham group (Fig. 3).

### The recovery pattern of normalized PPT values (*n* = 75)

**Fig. 4:**
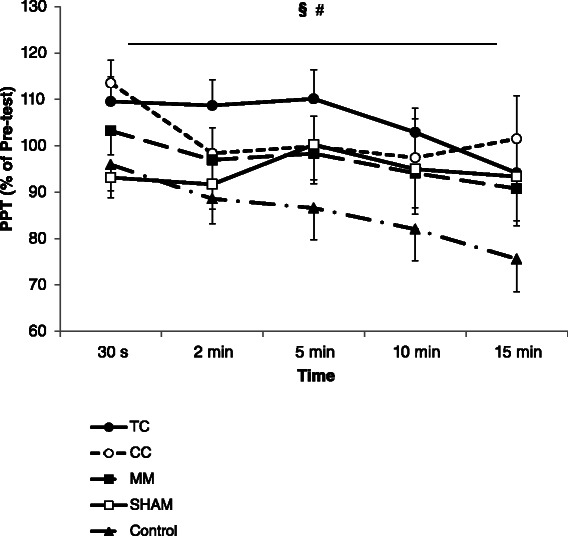
Pressure pain threshold (PPT) values (mean and SE) depicted as a percentage of pre-intervention value for each intervention group (*n* = 75, 15 per group) up to 15 min post-intervention. Ipsi-R: heavy rolling massage on the calf that exhibited the higher tenderness, Contra-R: heavy rolling massage on the calf of the contralateral limb, Sham: light stroking of the skin with roller massager on the calf that exhibited the higher tenderness, Ipsi-M: manual massage on the calf that exhibited the higher tenderness, and Control: no intervention. # denotes significant main effects for five time points. § denotes significant main effect for five interventions

A two-way ANOVA demonstrated significant effects for five time points (F = 2.6, *p* = 0.04) and five interventions (F = 6.6, *p* < 0.001). Post hoc analysis demonstrated that (regardless of time) PPT values were significantly higher following deep tissue massages including Ipsi-R (MD = 19.4 ± 4.1 % *p* < 0.001, ES = 0.83), Contra-R (MD = 16.4 ± 4.0 %, *p* = 0.001, ES = 0.69) and Ipsi-M (MD = 10.5 ± 4.1 %, *p* = 0.05, ES = 0.38) compared with the Control group (Fig. 4). In addition, (regardless of the intervention) PPT was significantly reduced over the time course of post-intervention measurements where 15 min post-intervention showed a statistically lower value than 30 s post-intervention (MD = −12.45 ± 4.2 %, *p* = 0.02, ES = 0.56) (Fig. 4). There was not a significant (*p* = 0.96) interaction effect of intervention × time.

## Discussion

The major findings of the study were that heavy rolling massage and manual massage over tender spots in plantar flexors increased the PPT compared with light rolling massage and control conditions. Interestingly, a similar effect was observed when heavy rolling massage was performed on the contralateral calf. The increased pain threshold however was transient and there was a significant decline of PPT (regardless of intervention effect) across 15 min post-intervention. Finally, when measuring pain via algometry, the algometer should be applied to a tender spot multiple times due to the change in PPT values that occur over multiple measurements.

We hypothesize that the rolling massage-induced decreases in pain could be due to mechanical stress or modulation of the central nervous system. Ipsilateral massage (Ipsi-R and Ipsi-M) may advocate an increased PPT via breaking up of fibrous adhesions and altering the response of free nerve endings (i.e. nociceptors) in the fascia [1, 2]. However, the non-localized effect of rolling massage on the contralateral limb suggest that other mechanisms such as a central pain-modulatory system may play a greater role in mediation of perceived pain following brief tissue massages.

The most plausible explanation we propose for the reduced pain perception in the present study could be the effect of heavy tissue massage on the central pain-modulatory systems [14, 34]. More specifically, massage-like mechanical pressure can provide analgesic effects via the ascending pain inhibitory system (gate theory of pain) [35, 36]. The activation of thick myelinated ergoreceptor nerve fibers (via activation of percutaneous mechanoreceptors and proprioceptors) can alter the transmission of ascending nociceptive information via small diameter Aδ fibers and give rise to a descending inhibitory effect that allows modulation of pain perception [7, 36]. The increase in PPT following heavy tissue massages in the ipsilateral and contralateral plantar flexors may be due, in part, to the mechanical pressure that rolling massage and manual massage exert on mechanoreceptor and proprioceptors. The effects of deep-tissue massage on perceived pain have been studied on various muscle groups. Kostopoulos and colleagues [37] demonstrated that ischemic compression massage significantly reduced perceived pain in trigger points located in the upper trapezius muscles. The same effect was observed when different combinations of ischemic compression, exercise and passive stretching were used on neck and shoulder muscles [38].

The second central nervous system pain-modulatory mechanism, which we propose to have contributed to improved pain perception in the present study, is the descending anti-nociceptive pathway (diffuse noxious inhibitory control (DNIC)) [34, 39]. DNIC, also known as counter-irritation is evoked by nociceptive stimuli (i.e. heat, high pressure, electrical stimulation) that ascends from the spinal cord to the brain. In turn, the brain inhibits pain transmission monoaminergically (i.e. using monoamine transmitters such as noradrenaline and serotonin) [34, 40], which leads to reduced pain perception not only locally but also at distant sites [40]. Our findings are in agreement with this theory because all three types of heavy tissue massage (i.e. high pressure) probably stimulated both skin and muscle nociceptors. In fact, the magnitude of pressure applied during the three deep tissue massages, either on ipsilateral (Ipsi-R and Ipsi-M) or contralateral limb (Contra-R), was adjusted to evoke a perception of pain equivalent to 7 out of 10 on VAS. Therefore, both the temporary increase of PPT and the observed effect following contralateral limb massage support the idea that the noxious counter-irritating mechanism might have been the contributing factor in mediation of pain perception following rolling and manual massage.

Third, we propose that parasympathetic reflexes controlled by the autonomic nervous system may contribute to the release of stress from myofascial tissue by relaxing/releasing/inhibiting the strain on the smooth muscles embedded in the soft tissue and subsequently increasing PPT. Massage has been shown to stimulate parasympathetic activities, which are characterized by the changes in biochemical substances such as serotonin, cortisol, endorphin and oxytocin [4]. On this basis, a potential explanation for the increased PPT following heavy tissue massages (Ipsi-R, Ipsi-M and Contra-R) could be an increase of parasympathetic activities and release of tension from myofascial tissue, which may release the noxious stimulus from free nerve endings (i.e. nociceptors). This explanation however remains speculative because the short-duration massages performed in the present study (i.e. 3 sets of 30 s of massages) resulted in a temporary increase of PPT. It could be postulated that if the parasympathetic-induced myofascial tissue property changes were the main mechanism contributing to modulation of pain, more persistent pain threshold alteration should have been observed from heavy massage. In line with our findings Vaughan and McLaughlin [14] demonstrated a temporary increase in PPT following 3 min of rolling massage, which was not present 5 min after the intervention. Although previous literature has indicated that massage may change microcirculation of blood flow, blood pressure, skin temperature and increase galvanic skin responses which all are indications of a lower level of sympathetic stimulation [4], there is no concrete evidence which shows that the effectiveness of massage is due to an increased blood flow, blood pressure and temperature.

Finally, we propose that massage-like mechanical stress that removes “trigger points” from muscle tissue may also lead to increased PPT. Myofascial trigger points are a common source of musculoskeletal pain [41, 42]. It is thought that application of massage-like mechanical pressure on trigger points can prevent the unnecessary firing of muscle spindles afferent discharges from the trigger point, reduce trigger point-induced muscle spasm and lead to decreased pain. There is however controversy about the identification and treatment of trigger points [43, 44]. In line with these debates, we are not certain if hypersensitive painful palpable taut bands identified in plantar flexor muscles in the present study were trigger points. In other words, the majority of tender spots identified in our study did not show the common criteria of being a trigger point (i.e. no local twitch response or referral pain pattern) [43]. Although hypersensitive taut bands in our study exhibited the signs of latent trigger points without noticeable twitch response and referral pain pattern [40], caution should be taken to interpret our finding as an evidence for effectiveness of rolling massage for trigger point therapy.

Interestingly, a decline in pain threshold was observed following light rolling massage. Previous investigations have indicated that the PPT value depends on the sensitivity of both superficial and deep tissues nociceptive receptors [21, 45]. It has also been suggested that the descending anti-nociceptive system has a greater influence on input from muscle nociceptors than skin nociceptors [46]. Since light rolling massage was not a noxious stimuli, the decreased PPT following this intervention may be associated with increased sensitivity of superficial nociceptors compared with heavy massage (Ipsi-R, Contra-R, Ipsi-M), which exerted noxious deep tissue pressure on the muscles and raised the pressure pain threshold. However more studies are required to support this hypothesis because pressure pain threshold may predominantly reflect muscle nociception and it may be less influenced by cutaneous analgesia [45].

Pain threshold measurement using pressure algometry has been suggested as a reliable measure to evaluate relative tenderness in healthy individuals [18, 20, 27, 28]. Previous investigations demonstrated high interclass correlation coefficient (between 0.80 and 0.97) for this measurement [19, 22, 23, 27, 28]. Several investigations have reported high reliability coefficients (range: 0.71–0.97) for 2 to 5 repeated PPT algometry trials over tender spots in various muscle groups [19, 22, 23]. In line with these findings, the ICC calculated for 6 pretest PPT trials (*n* = 150) in the present study was 0.93, which showed an excellent reliability for repetitive pressure algometry (with 5–10 s time interval between trials). However, it should be noted that ICC value is sensitive to between-subject variability [30]. The ICC increases with increasing CV [18, 47]. Thus, the high ICC value observed in our study could be due to the large CVs that we found for the 6 pre-intervention PPT trials (~46 %). Considerable inter-individual variability for PPT measurement across subjects has been previously reported in literature [28, 48, 49]. Therefore, in order to confirm the reliability of our PPT measurements, we also analyzed the differences between group means. Interestingly, our data demonstrated a significant decline in pain threshold across six algometry trials where the 3^rd^, 4^th^, 5^th^ and 6^th^ trials showed significantly lower threshold than the 1^st^ and 2^nd^ PPTs, which did not occur following the intervention. A reduction in PPT values has been indicated as mechanical hyperalgesia [5]. In other words, the current results indicate that the first two PPT trials may have caused a generalized state of increased sensitization of the nociceptors. In line with our finding Wolff and Jarvik [24] suggested to discard the first trial of pain threshold measurement and use the average of at least 5 trials for heat, cold and chemical stimulations. Other studies have also indicated that to increase between-sessions reliability, the average of at least 2–3 trials should be used [19, 23]. This is the first investigation that reveals the significant influence of repetitive pressure algometry on pain thresholds obtained from hypersensitive tender spots in plantar flexor muscles. These findings uncover the responses of repetitive pressure pain algometry applied to a hypersensitive tender spots and provides insight about the clinical application of pressure algometry on pathological degree of tenderness.

### Study limitations

There are several limitations in the study. 1) Participants in the present study undertook 3 sets of 30s rolling massage. The duration of massage may not have been enough to produce greater and longer changes in PPT. Therefore, more research is required to ascertain the optimal rolling massage duration for increased PPT. 2) We measured the effect of only one session of rolling massage and with a short follow up period (15 min) whereas further studies are required to investigate the cumulative effect of using roller massage on PPT. 3) Participants in the present study were volunteers, this may introduce a bias because individuals who take part in a massage intervention are likely to believe in the benefits of the therapy. Therefore the Sham and Control intervention groups were recruited to monitor any potential effect. 4) In the present study, the effect of different types of massage was not measured on sex differences due to a small sample size. Riley et al., [33] suggested that a minimum of 41 subjects per group was required for studying the gender effects. 5) All participants in our study were university-aged individuals; therefore more research with other ranges of age groups is necessary. 6) The pathology of the plantar flexors muscle pain in the present study was limited to existence of trigger points; thus more studies are required to determine the effect of rolling massage on pain modulation with other pathology of musculoskeletal pain.

## Conclusions

In conclusion our findings suggest that heavy rolling massage on muscles containing a hypersensitive tender spot (Ipsi-R) can provide an acute increase in pain threshold. Similar effect can be observed when heavy rolling massage is performed on contralateral muscle group (Contra-R). Since the increase in pain threshold shows a transient and non-localized effect, it could be postulated that central pain-modulatory system may play the main role in mediation of perceived pain following brief rolling massages. Our results also suggest that when measuring pain via algometry, it is important to measure PPT multiple times due to participants overestimating PPT in the early measurements. PPT measurements thereafter are reliable.

## Abbreviations

PPT: Pressure pain threshold
Ipsi-R: Ipsilateral rolling massage
Contra-R: Contralateral rolling massage
Ipsi-M: Ipsilateral manual massage
DOMs: Delayed onset muscle soreness
VAS: Visual analogue scale
ICC: Intra-class coefficient
CV: Coefficient of variation
ES: Effect size
MD: Mean difference
DNIC: Diffuse noxious inhibitory control
